# A single giant frontoparietal intraaxial hydatid cyst in a 5-year-old female: a case report and review of literature

**DOI:** 10.1093/omcr/omae180

**Published:** 2025-01-18

**Authors:** Alaa R AL-Ihribat, Yaman N Qunaibi, Rawand B Amro, Jamal Ahmed, Rama F Rije, Husam Shalalfeh, Falah Ibedo, Saeed Itkaidek

**Affiliations:** College of Medicine and Health Sciences, Palestine Polytechnic University, Wadi Al-Hariyah Street, Hebron, 00970, Palestine; College of Medicine and Health Sciences, Palestine Polytechnic University, Wadi Al-Hariyah Street, Hebron, 00970, Palestine; College of Medicine and Health Sciences, Palestine Polytechnic University, Wadi Al-Hariyah Street, Hebron, 00970, Palestine; College of Medicine and Health Sciences, Palestine Polytechnic University, Wadi Al-Hariyah Street, Hebron, 00970, Palestine; College of Medicine and Health Sciences, Palestine Polytechnic University, Wadi Al-Hariyah Street, Hebron, 00970, Palestine; Neurosurgery Department, Al-Ahli Hospital, Hebron, 00970, Palestine; College of Medicine and Health Sciences, Palestine Polytechnic University, Wadi Al-Hariyah Street, Hebron, 00970, Palestine; Anesthesia Department, Al-Ahli Hospital, Hebron, 00970, Palestine; Neurosurgery Department, Al-Ahli Hospital, Hebron, 00970, Palestine

**Keywords:** Echinococcus granulosus, hydatid cyst, cerebral cyst, frontoparietal hydatid cyst

## Abstract

Echinococcus larval stage or a hydatid cyst, a parasitic disease that passes from animals to humans. Echinococcus granulosus and, less commonly, Echinococcus multilocularis species cause the disease. Intracranial echinococcosis is rare, with an incidence of approximately 1%–2%. Primary and secondary intracranial hydatid cysts can be found. A primary one, the most common type, is always solitary. CT and MRI have significantly contributed to the accurate diagnosis of hydatids. Surgical removal of the cyst is the treatment of choice, resulting in most cases in a complete recovery. In this report, we aim to emphasize the presentation of such an isolated primary cerebral hydatid cyst in a 5-year-old girl with neurological symptoms of a space-occupying lesion. Radiology revealed a giant left frontoparietal hydatid cyst. A cerebral hydatid cyst was operated on. The patient showed an uneventful recovery and was discharged. There was no recurrence at follow-up.

## Introduction

A hydatid cyst is a parasitic disease most commonly caused by Echinococcus granulosus, followed by Echinococcus multilocularis. Humans acquire the infection by accidentally ingestion eggs that are transported to the liver through the intestine and portal system. Humans as hosts of Echinococcus granulosus may experience organ involvement in various parts of the body, most commonly the liver and rarely the lung, muscles, heart, spine, and brain. Central nervous system involvement by a hydatid cyst is rare (1%–2% of all cases) and may be located anywhere in the brain, most often in the parietal lobe, followed by the frontal lobe. Rarely, cysts are located in the posterior cranial fossa or ventricles. Brain hydatid cysts do not produce symptoms until they reach a significant size [1]. Symptoms include increased intracranial pressure (ICP), due to location and mass effect of the hydatid cyst in the central nervous system, also, cranial nerve paralysis may develop. The best treatment modality is surgery, and the aim of the surgery is total extirpation of the cyst without rupturing the cyst wall [2].

## Case presentation

**Figure 1:**
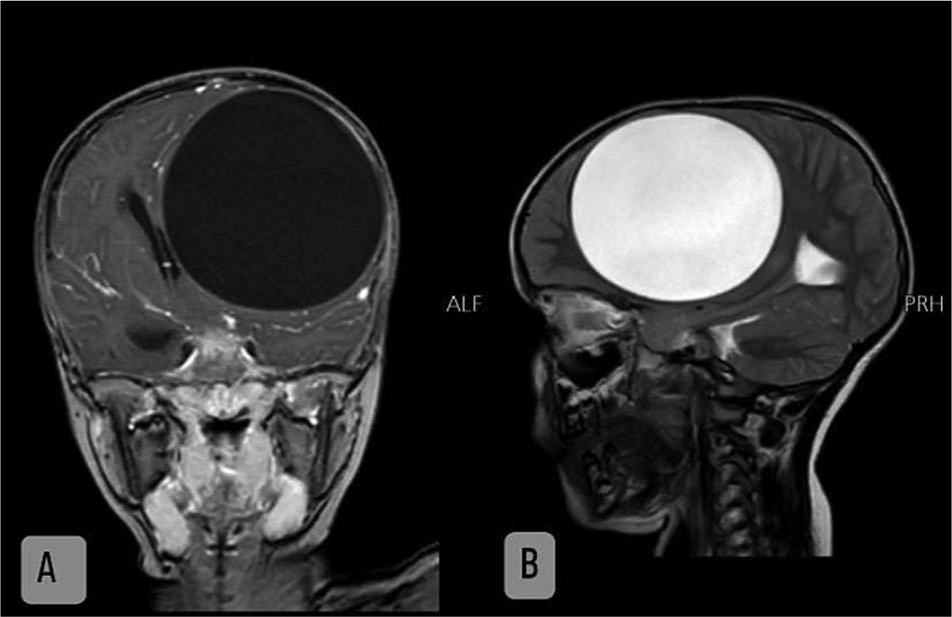
Coronal (**A**) and sagittal (**B**) view MRI shows intra-axial cystic lesion measuring 10 × 8 × 9 cm lies at left frontoparietal area, cyst fluid is isointense with CSF.

A 5-year-old girl with no significant past medical history was referred from an outpatient clinic. She had been healthy until three months prior when she began experiencing generalized weakness, fever, and hypoactivity. Despite multiple medical consultations and treatments for upper respiratory infections and flu, her symptoms persisted. Two months ago, she was clinically diagnosed with meningitis in an outpatient clinic without confirming diagnosis by imaging or CSF analysis, treated with empirical intravenous antibiotics, and showed some improvement. However, within two weeks, she became hypoactive again and developed morning headaches, generalized weakness, limb weakness, frequent urination, and mouth deviation. An initial brain CT scan revealed a large left frontoparietal intraaxial cystic lesion measuring 10 × 8 × 9 cm with a midline shift. An MRI confirmed the presence of a large intraaxial cyst (Fig. 1).

The patient was referred to our neurosurgical department for surgery. Thoracic and abdominal CT scans were performed to identify other cystic lesions in the liver, spleen, or lungs, but the results were unremarkable.

**Figure 2:**
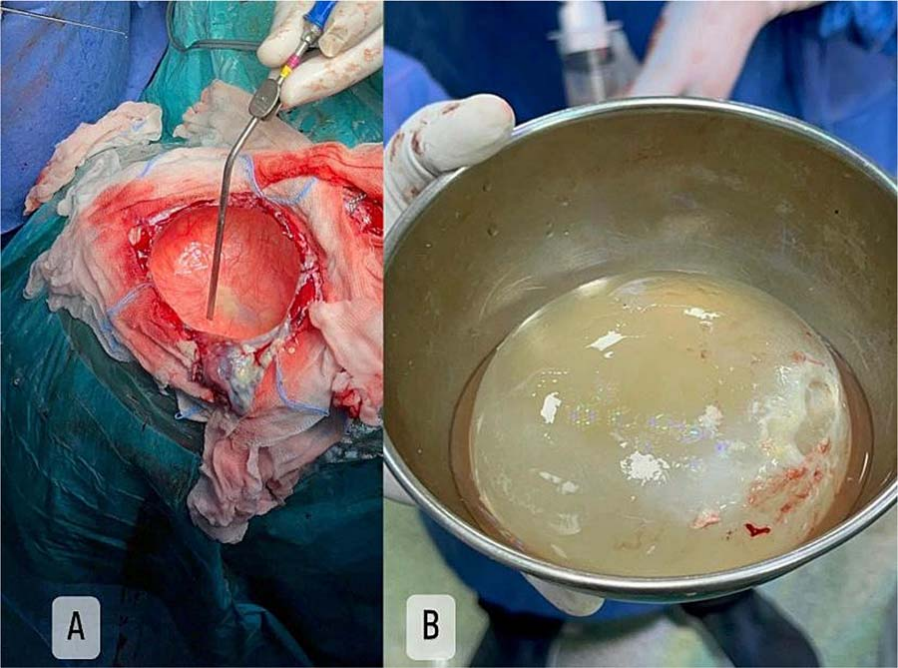
Intra-operative craniotomy (**A**) with excision of intact cyst (**B**).

**Figure 3:**
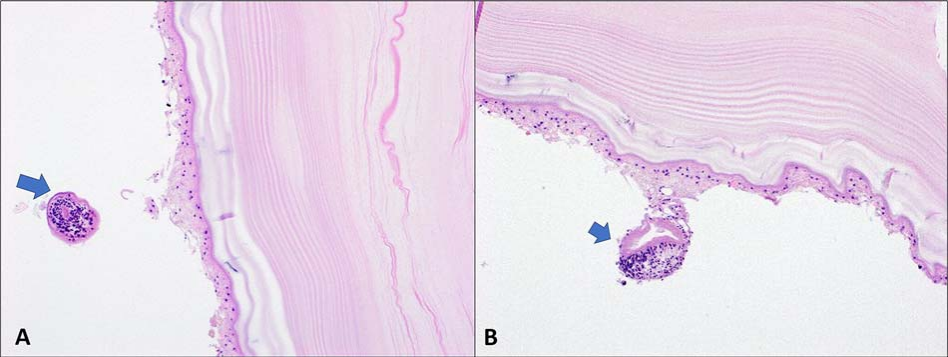
Echinococcal cyst due to *E. granulosus*; (**A** and **B**). Laminated acellular membrane is noted with presence of protoscolices near/attached to the internal surface (arrows). (H&E, 20X).

Craniotomy and excision of the cyst without rupture were performed (Fig. 2). The pathology report confirmed a hydatid cyst with multiple protoscolices (Fig. 3). A follow-up CT showed a bone defect in the left front-parietal–temporal region with pneumocephalus, consistent with postoperative changes. The patient was discharged a few days later in good general condition.

## Discussion

Echinococcus is a cestode whose larval stage causes illness in humans. It can present in several forms. Cystic echinococcosis (CE), caused by Echinococcus granulosus, leads to hydatid cyst formation. Alveolar echinococcosis (AE), linked to Echinococcus multilocularis, behaves like a malignant tumor. Polycystic echinococcosis involves multiple cysts. Rarely, cyst rupture can occur, which may cause anaphylaxis. Dogs and foxes are the definitive hosts, which carry the adult parasite, while sheep, goats, and cattle are the intermediate hosts which harbor the larval stage. Infection doesn’t always require direct contact with dogs, as consuming food, water, or milk contaminated with tapeworm eggs can lead to infection. The liver is the most common organ affected, followed by the lungs. Other organs, including those of the central nervous system, are less frequently involved [3].

Brain hydatid cysts represent 1%–2% of all cases. Children are more likely to develop these cysts due to their open sutures, flexible skull bones, and soft brain tissue. Studies have shown a slight male predominance. These cysts grow faster in soft tissues like the brain and liver [2], typically expanding by 1–5 cm annually. In contrast, the cyst discovered in our patient was exceptionally large, measuring 10 cm in diameter, and occupied nearly the entire left cerebral hemisphere.

Brain hydatid cysts can be categorized as either primary (single) or secondary (multiple). Primary cysts develop directly in the brain without affecting other organs. Secondary cysts, on the other hand, arise from the spontaneous, accidental, or surgical rupture of a solitary brain cyst [4]. In this particular instance, since cysts were detected exclusively in the brain, it can be concluded that the brain was the primary site of hydatid cyst development.

Symptoms depends on cyst’s location, size, number, and the patient’s immune response. Children with brain cysts may show symptoms of increased intracranial pressure like headaches, seizures, nausea, vomiting, papilledema, and facial nerve paralysis. Less commonly, they may experience balance and coordination problems, and sluggishness [5]. Our patient’s neurological status at presentation was described as having morning headaches, decreased activity, weakness in arms and legs, frequent urination, and mouth drooping.

CT and MRI are used to diagnose intracranial cysts. Solitary cysts typically appear as well-defined, low- attenuating, round lesions with smooth, thin, non-enhancing wall after contrast injection. These cysts may compress the brain’s ventricular system, leading to hydrocephalus and brain herniation [6]. Serological tests, which detect specific *E. granulosus* antigens, are commonly used to confirm imaging results [4].

Surgery is the primary treatment, supplemented by albendazole. Albendazole helps kill the cyst, reducing the risk of anaphylactic shock, and prevent recurrence [7]. Dowling’s hydrodissection is a commonly used surgical approach. This involves gently washing the area between the cyst and the brain with saline solution to remove the cyst intact. This is often feasible due to limited scar tissue around the cyst [8]. This procedure was successfully performed in our case, and albendazole treatment was initiated upon admission.

Among review of literatures, there were only two reported cases of cerebral hydatid cysts at this age (Fakhouri et al., [8], and Bahloul et al., [9]). One case involved a cyst in the posterior fossa and the other in the frontoparietal region; both patients experienced seizure as initial complaint, unlike our patient. The symptoms our patient exhibited complicated the diagnosis. However, with the aid of radiological imaging, an accurate diagnosis and appropriate treatment were provided.

## Conclusion

Intracranial hydatid cysts should be surgically removed without rupture to prevent recurrence and anaphylactic shock. Accurate preoperative diagnosis is essential for the surgical success. Medical treatment with albendazole seems to be beneficial both before and after surgery.

## Data Availability

The data that support the findings of this study are available from corresponding author upon reasonable request.
